# Cost-effectiveness analysis of pembrolizumab plus chemotherapy versus chemotherapy as first line chemotherapy for patients with unresectable advanced esophageal cancer in Japan

**DOI:** 10.1007/s10388-025-01144-5

**Published:** 2025-07-12

**Authors:** Hirohito Kakinuma, Daisuke Takada, Hisashi Itoshima, Susumu Kunisawa, Kensuke Moriwaki, Michitaka Honda, Kiyohide Fushimi, Yuichi Imanaka

**Affiliations:** 1 Department of Healthcare Economics and Quality Management, School of Public Health, Kyoto University Graduate School of Medicine, Kyoto, Japan; 2 Department of Minimally Invasive Surgical and Medical Oncology, Fukushima Medical University, Fukushima, Japan; 3 Department of Surgery, Southern Tohoku Research Institute for Neuroscience Southern Tohoku General Hospital, Fukushima, Japan; 4 Department of Food Science and Nutrition, Faculty of Human Life and Science, Doshisha Women’s College of Liberal Arts, Kyoto, Japan; 5 Comprehensive Unit for Health Economic Evidence Review and Decision Support (CHEERS), Research Organization of Science and Technology, Ritsumeikan University, Kyoto, Japan; 6 Division of Health Service Research, Department of Biomedical Science, College of Life Sciences, Ritsumeikan University, Kyoto, Japan; 7 Department of Health Policy and Informatics, Institute of Science Tokyo Graduate School, Tokyo, Japan; 8 Department of Health Security System, Centre for Health Security, Kyoto University Graduate School of Medicine, Yoshida Konoe‐cho, Sakyo‐ku, Kyoto, 606‐8501 Japan

**Keywords:** Esophageal neoplasms, Antineoplastic agents, Cost-effectiveness analysis

## Abstract

**Background:**

Although pembrolizumab plus chemotherapy (Pembro + Chemo) is the recommended first-line therapy for advanced esophageal cancer, it remains unclear whether it is more cost-effective than chemotherapy (Chemo). We evaluated the cost-effectiveness of Pembro + Chemo versus Chemo from a Japanese healthcare payer perspective.

**Methods:**

A partitioned survival analysis model was developed to predict costs and quality-adjusted life years (QALY) for Pembro + Chemo and Chemo. The model parameters were based on a previous randomized controlled trial and a nationwide administrative database in Japan. The incremental cost-effectiveness ratio (ICER) of Pembro + Chemo compared with Chemo was estimated. A subgroup analysis on the level of PD-L1 Combined Positive Score (CPS) ≥ 10 was also conducted. Additionally, one-way deterministic sensitivity analysis and probabilistic sensitivity analysis with Monte Carlo simulations (5,000 simulations) were conducted.

**Results:**

The ICER of Pembro + Chemo over Chemo was estimated at USD 176,479 per QALY. The subgroup analysis for patients with PD-L1 CPS ≥ 10 showed an ICER of USD 126,862 per QALY. One-way deterministic sensitivity analysis demonstrated that the ICER remained above the willingness-to-pay threshold of USD 50,000–100,000 per QALY in all scenarios, with the utility of progression-free survival in the Pembro + Chemo group having the greatest influence on the ICER. Probabilistic sensitivity analysis showed that the probability of Pembro + Chemo being cost-effective was 0% at the willingness-to-pay threshold.

**Conclusion:**

The addition of pembrolizumab to chemotherapy was not cost-effective in treating advanced esophageal cancer in Japan.

**Supplementary Information:**

The online version contains supplementary material available at 10.1007/s10388-025-01144-5.

## Introduction

Esophageal cancer is a major global health problem because it has a high incidence rate and quickly advances, leading to high mortality rates. Among all cancers worldwide, esophageal cancer ranks 11th in incidence (511,054 cases) and 7th in mortality (445,391 deaths) annually [1]. Treatment strategies for unresectable advanced esophageal cancer have evolved over time. Historically, the standard first-line treatment was combined fluoropyrimidine plus platinum-based chemotherapy (FP therapy) [2, 3]. Recently, adding pembrolizumab to FP therapy (Pembro + FP therapy) was found to improve patient outcomes. This is based on the results of the KEYNOTE-590 trial, an international phase III trial, which demonstrated that Pembro + FP therapy improved patient survival compared with FP therapy alone [4]. Based on this finding, the Esophageal Cancer Practice Guidelines 2022 recommend Pembro + FP therapy as first-line therapy for treating patients with unresectable advanced esophageal cancer [5].

Cost-effectiveness evaluations of Pembro + FP therapy have varied between countries. While studies from the United States and the National Institute for Health and Care Excellence (NICE) in the United Kingdom have demonstrated favorable cost-effectiveness [6, 7], economic evaluations in China have been less favorable [8]. These contradictory findings may reflect differences in national healthcare systems, such as drug access, that may have contributed to the cost-effectiveness differences between the countries. Japan, like South Korea and Taiwan, operates under a universal health insurance system with free access to healthcare services [9–11]. Moreover, the unique drug pricing system of Japan has established one of the lowest prices for pembrolizumab globally [12]. However, a cost-effectiveness analysis of Pembro + FP therapy has not been conducted in Japan or other countries with similar universal healthcare systems.

More than 70% of esophageal cancer patients are concentrated in Asia [1], with Japan reporting approximately 24,000 new cases and 10,000 deaths annually [13]. Given the substantial disease burden in Asian countries, it is crucial that a cost-effectiveness analysis be conducted using real-world data from Japan that reflects clinical practice. Therefore, this study aimed to evaluate the cost-effectiveness of Pembro + FP therapy for unresectable advanced esophageal cancer using real-world data from Japan.

## Method

### Patient population, intervention, and comparators

The analysis targeted a hypothetical cohort of Japanese patients with unresectable advanced esophageal cancer based on the patient characteristics of the KEYNOTE-590 trial [4]. In accordance with the KEYNOTE-590 trial, Pembro + FP therapy was selected as the intervention group, and FP therapy was the control group [4]. Prior to the approval of pembrolizumab in Japan, FP therapy was the standard treatment [5]. Although treatment regimens combining 5-FU with paclitaxel, irinotecan, capecitabine have been reported, their clinical positioning remains unclear, and these regimens are not recommended in the Clinical Practice Guidelines of the Japan Esophageal Society [5]. Therefore, selecting FP therapy as the comparator treatment was considered appropriate.

### Model-based cost-effectiveness analysis

We conducted a model-based cost-effectiveness analysis to evaluate pembrolizumab plus FP therapy versus FP therapy as first-line treatment for unresectable advanced esophageal cancer from the Japanese healthcare payer perspective. To predict long-term costs and quality-adjusted life years (QALYs), we constructed a partitioned survival analysis (PartSA) model, the standard approach for anticancer drug cost-effectiveness analyses [14]. The model represented the prognosis of patients with cancer through three health states: progression-free survival (PFS), progressed disease (PD), and death. To estimate the temporal distribution of patients across health states, we fitted parametric functions to the Kaplan–Meier curves for PFS and overall survival (OS) from the KEYNOTE-590 trial [15]. Long-term costs and QALYs were calculated by multiplying the proportion of patients in each health state by state-specific costs and utility values per cycle. The Incremental Cost-Effectiveness Ratio (ICER) was calculated using the following formula: ICER = (Cost _Pembro+FP_—Cost _FP_)/(QALY _Pembro+FP_—QALY _FP_). The willingness-to-pay (WTP) threshold was set between 50,000 and 100,000 USD (7.5 to 15 million yen) per QALY in accordance with the Japanese Health Technology Assessment (HTA) drug pricing system criteria [16]. We used the upper limit of the reference value, 100,000 USD (15 million yen), as the threshold value, but its validity is not clear. Therefore, future research on Japan’s WTP is needed. The time horizon was set to 38 years, with an annual discount rate of 2% applied to long-term costs and health-related outcomes, following the Cost-effectiveness Evaluation Guidelines of the Central Social Insurance Medical Council [17]. The model cycle length was set to one month. Additionally, we analyzed subgroup data for PD-L1 Combined Positive Score (CPS) ≥ 10 from the KEYNOTE-590 trial [4]. The model was developed and analyzed using TreeAge Pro^®^ 2023 (TreeAge Software LLC, Williamstown, MA, USA).

### Curve fitting

To estimate the PFS and OS for each treatment group required for PartSA model simulation, we performed parametric curve-fitting on the Kaplan–Meier curves from the KEYNOTE-590 trial [15]. While curve fitting usually requires individual patient survival data from a clinical trial, such data was not publicly available for the KEYNOTE-590 trial. Therefore, we employed an alternative approach. Using the methodology developed by Guyot et al., we reconstructed individual patient survival data from the Kaplan–Meier curve images and the number at risk data [18]. First, we extracted time point and cumulative survival probability information from the Kaplan–Meier curve images of the KEYNOTE-590 trial. Second, using this extracted information and the number of patients at risk data from the KEYNOTE-590 trial, we generated pseudo-individual patient survival data following Guyot’s method. We used WebPlotDigitizer (Ankit Rohatgi, Pacifica, CA) for extracting information from the images and R (R Foundation for Statistical Computing, Vienna, Austria) for creating individual patient data, constructing individual survival times from both OS and PFS Kaplan–Meier curves. For curve fitting, we applied six types of parametric functions: exponential, Weibull, log-normal, log-logistic, Gompertz, and generalized gamma. The analysis was conducted using Stata BE 18.5 (Stata Corp LLC. College Station, TX, USA). Selection of the optimal function was based on a comprehensive visual evaluation, Akaike Information Criterion (AIC), Bayesian Information Criterion (BIC), and clinical validity (Supplementary Table 1). Based on this evaluation, we adopted the log-logistic function for both OS and PFS curves in both the base case and subgroup analyses (Supplementary Table 2).

### Costs

This study evaluated direct medical costs from the perspective of Japanese healthcare payer. All costs were calculated in Japanese yen and converted to US dollars using an exchange rate of 1 USD = 150 JPY. In this model, the following cost parameters were set: (1) drug costs (per month) before disease progression in each group; (2) other medical costs (per month) before disease progression in each group; (3) total medical costs (per month) after disease progression in each group; (4) end-of-life medical costs (per case).

For the Pembro + FP therapy group, we assumed a treatment regimen of pembrolizumab 200 mg (day 1), cisplatin 80 mg/m^2^ (day 1), and 5-fluorouracil 800 mg/m^2^ (days 1–5) every three weeks. The dosage was determined based on the average body surface area of Japanese patients, calculated using the gender distribution of the KEYNOTE-590 trial and national health survey data [19]. Total medical costs after disease progression were assumed to be identical for both groups. Drug costs were calculated using Japan’s drug price standards [20]. While the Ministry of Health, Labour and Welfare’s Optimal Use Guidelines for Pembrolizumab in Esophageal Cancer does not specify an upper limit for pembrolizumab administration duration [21], the KEYNOTE-590 trial limited it to 2 years [4]. Therefore, we treated Pembro + FP therapy limited to 2 years as the base case analysis and when no upper limit was set on the duration of treatment as the sensitivity analysis.

The medical cost parameters were estimated using the Diagnosis Procedure Combination (DPC) database managed by the DPC research group supported by the Ministry of Health, Labour and Welfare. The DPC per-diem payment system (DPC/PDPS) is a prospective payment system for acute inpatient care in Japan, involving over 1700 acute care hospitals, including all university hospitals [22]. The database represents approximately 54% of general hospital beds nationwide and covers the majority of short-term inpatients [23]. The DPC data comprises clinical summary information, insurance claims, facility identifiers, discharge status (alive or in-hospital death), primary diagnosis, most and second-most resource-intensive diagnoses, up to 10 comorbidities, and up to 10 complications. It includes medical costs, procedure dates, frequency and volume of medical procedures (techniques and tests), prescribed medications, and medical materials. Diagnostic information is coded according to the International Classification of Diseases, 10th revision (ICD-10). The DPC data is used to reimburse acute care hospitals under the public health insurance system and for research to improve healthcare quality. The analysis period was from December 1, 2021, to August 31, 2024. We assumed that all chemotherapy before disease progression was administered during hospitalization, while all chemotherapy after disease progression was administered in outpatient settings, so we calculated these costs separately.

Patients were included in the cost analysis before disease progression if they had esophageal cancer (ICD-10 codes: C150, 151, 152, 154, 155, 159). They were subdivided into two groups: the Pembro + FP group included patients who received pembrolizumab, fluorouracil, and cisplatin treatment during hospitalization; the FP group included patients who received fluorouracil and cisplatin treatment during hospitalization. The following exclusion criteria were applied: (1) Pembro + FP group: cases not using all three drugs (pembrolizumab, fluorouracil, and cisplatin), (2) FP group: cases not using both drugs (fluorouracil and cisplatin), and (3) Cases with hospitalization periods of less than 5 days or more than 11 days (this criterion was established because the regimen requires a 5-day fluorouracil administration period, and the average length of stay for target cases in the DPC data was approximately 7 days).

Cost analysis after disease progression included esophageal cancer patients (ICD-10 codes: C150, 151, 152, 154, 155, 159) who received outpatient treatment with either pembrolizumab, nivolumab, or paclitaxel. Terminal care costs were defined as medical costs in the month before death for cases that received additional fees for palliative care and did not undergo anticancer drug treatment.

### Utility weights

Table 1 shows the PFS and PD utility values. Without utility value data for Japanese esophageal cancer patients, we used alternative data obtained from the ToGA trial as a substitute [24, 25]. The ToGA trial was an international phase III clinical study that evaluated the efficacy of adding trastuzumab to standard chemotherapy in HER2-negative patients with unresectable advanced gastric cancer [24]. In this study, we cited utility values from a previous study (Shiroiwa et al.), which conducted a health economic evaluation using data from the ToGA trial [26]. Their study calculated utility values using EQ-5D data collected in the ToGA trial, which were converted using a Japanese algorithm. We selected the utility values derived from the ToGA trial for two main reasons. First, these values have been used in previous cost-effectiveness studies of advanced esophageal cancer [8, 27]. Second, the availability of Japanese-specific utility data from the ToGA trial makes these values more applicable to our study population.

**Table 1:** Parameter setting

Item	Estimate	Plausible range		Distribution	References
Cost parameters					
Drug treatment costs before PD					
Pembrolizumab (USD per month)	4,153.77	− 10%	10%	Triangle	[10]
FP (USD per months)	136.11	− 10%	10%	Triangle	[10]
Other medical costs before PD					
Pembrolizumab + FP (USD per month)	2,839.79	2,753.38	2,926.21	Gamma	DPC
FP (USD per month)	1,438.60	1,392.67	1,484.55	Gamma	DPC
Medical costs after PD (USD per month)	1,134.05	1,130.41	1,137.68	Gamma	DPC
Terminal care costs (USD per cases)	9,549.11	9,471.75	9,626.49	Gamma	DPC
Utility before PD					
Pembrolizumab + FP	0.815	− 10%	10%	Triangle	[14]
FP	0.797	− 10%	10%	Triangle	[14]
Utility after PD	0.577	− 10%	10%	Triangle	[14]
Other settings					
Discount rate (%)	2	0	4		[8]
Body weight of hypothetical patients (kg)	65.1				[17]
Body height of hypothetical patients (cm)	165.5				[17]
Duration of Pembrolizumab	2	2	38		[19]
Time horizon (year)	38				[8]

### Sensitivity analysis

We conducted one-way sensitivity analyses to evaluate the robustness of the base-case analysis. The ranges for cost parameters were established based on the 95% confidence intervals obtained from the DPC database (Table 1). We set variation ranges for drug prices and utility values at ± 10% of the base-case estimates. The results of the sensitivity analyses were presented using tornado diagrams. Additionally, we performed supplementary sensitivity analyses assessing the effect of parametric function selection for OS and PFS on the results.

To quantify the uncertainty in cost-effectiveness, we conducted a probabilistic sensitivity analysis using second-order Monte Carlo simulation (5000 simulations). Table 1 shows the probability distributions for each parameter used in the probabilistic sensitivity analysis. Appropriate probability distributions were applied to each parameter based on the variability of available data. In each of the 5000 simulations, the value for each model input was randomly selected from its distribution. From the second-order Monte Carlo simulation, we generated a cost-effectiveness acceptability curve and estimated the probability of Pembro + FP being cost-effective at WTP thresholds ranging from 50,000 to 100,000 USD/QALY.

## Results

### Base case analysis

**Fig. 1:**
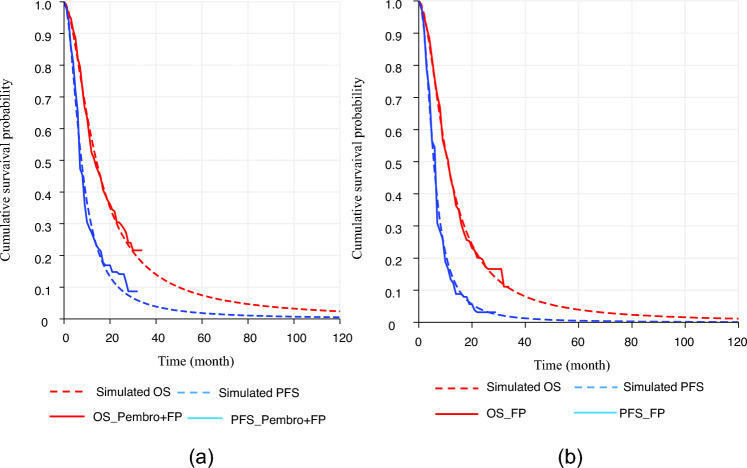
Results of Survival curves. **a** OS and PFS in Pembro + FP group **b** OS and PFS in FP group. *Pembro* + *FP* pembrolizumab plus FP therapy, *FP* fluoropyrimidine plus platinum-based chemotherapy, *PFS* progression-free survival, *PD* progressive disease

Figure 1 shows the curve-fitting results for PFS and OS, and Table 2 presents the results of the base case analysis. The PFS was 12.67 months in the Pembro + FP group and 8.53 months in the FP group. Post-progression survival was 12.54 months in the Pembro + FP group and 9.85 months in the FP group. The QALYs were 1.38 for the Pembro + FP group and 1.00 for the FP group, resulting in an incremental QALY of 0.38 for the Pembro + FP group. Cost analysis showed total costs of $99,975 for the Pembro + FP group and $32,866 for the FP group. Based on these results, the ICER of Pembro + FP compared to FP was $176,479/QALY.

**Table 2:** Base case and subgroup analyses

	Survival (month)	LY	Incremental LY	QALY	Incremental QALY	Cost	Incremental cost	ICER
	PFS PD					(USD)	(USD)	(USD/QALY)
Overall (base case)								
Pembrolizumab + FP	12.67 12.54	2.10	0.57	1.38	0.38	99,975	67,109	176,479
FP	8.53 9.85	1.53		1.00		32,866		
PD-L1 CPS 10 or more								
Pembrolizumab + FP	13.74 13.50	2.27	0.87	1.48	0.58	103,741	73,183	126,862
FP	7.09 9.68	1.40		0.90		30,557		

*PFS* progression-free survival, *PD* progressive disease, *LY* life year, *QALY* quality—adjusted life year, *ICER* incremental cost-effectiveness ratio, *FP* fluoropyrimidine plus platinum-based chemotherapy

### *Subgroup analysis (PD-L1 CPS* ≥ *10)*

Supplementary Fig. 1 shows the curve-fitting results for PFS and OS, and Table 2 presents the results of the subgroup analysis. In the subgroup analysis, the Pembro + FP group showed a PFS of 13.74 months, post-progression survival of 13.50 months, and OS of 27.24 months. In contrast, the FP group demonstrated a PFS of 7.09 months, post-progression survival of 9.68 months, and OS of 16.77 months. Total costs were $103,741 for the Pembro + FP group and $30,557 for the FP group, with an incremental cost of $73,183. The QALYs were 1.48 for the Pembro + FP group and 0.90 for the FP group, yielding an incremental QALY of 0.58. The resulting ICER was $126,862/QALY. Although this ICER was lower than in the base case analysis, it still exceeded the WTP threshold.

### Sensitivity analysis

**Fig. 2:**
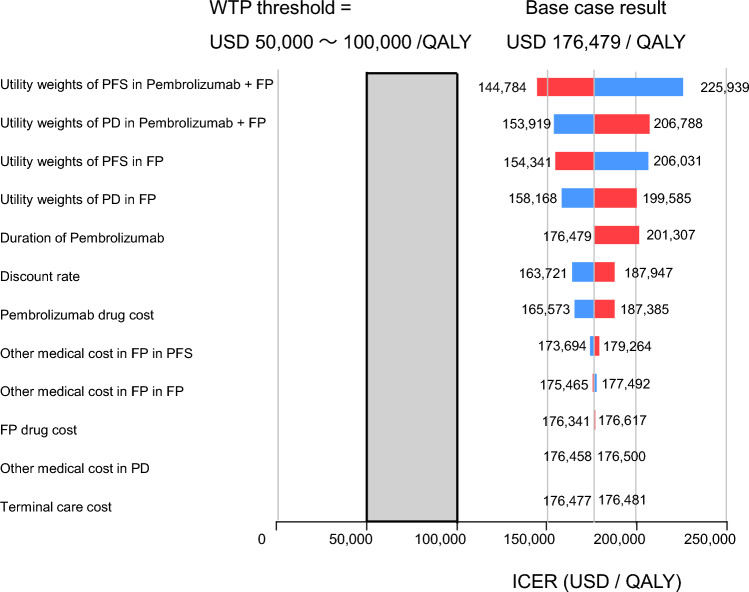
Results of Deterministic sensitivity analyses. *Pembro* + *FP* pembrolizumab plus FP therapy, *FP* fluoropyrimidine plus platinum-based chemotherapy, *PFS* progression-free survival, *PD* progressive disease, *QALY* quality-adjusted life year

**Fig. 3:**
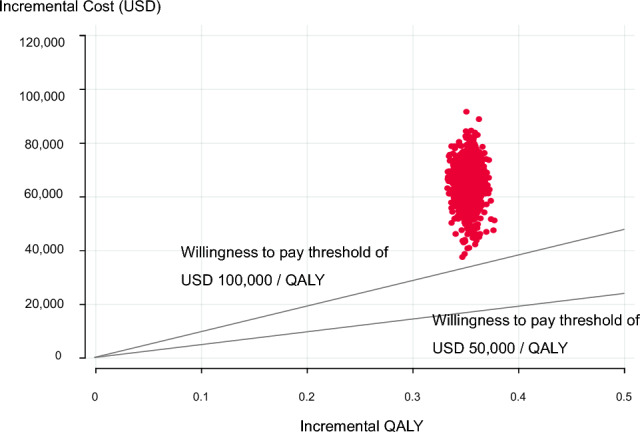
Results of Probabilistic sensitivity analyses (Scatter plot)

**Fig. 4:**
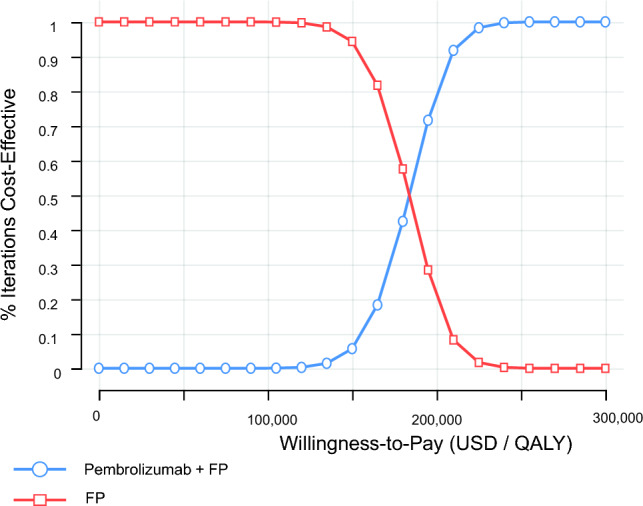
Cost-effectiveness acceptability curve. *Pembro* + *FP* pembrolizumab plus FP therapy, *FP* fluoropyrimidine plus platinum-based chemotherapy

Figure 2 presents the one-way sensitivity analysis results as a tornado diagram. The utility of PFS in the Pembro + FP group had the greatest impact on the ICER. When this utility was at its highest, the ICER was estimated to be $144,785/QALY, and at its lowest, $225,939/QALY. However, neither case fell below the WTP threshold of $50,000–$100,000/QALY. Table 3 shows the scenario analyses for curve fitting. The log-logistic function used in the base analysis yielded the most favorable ICER, while the Gompertz function yielded the highest ICER. Figures 3 and 4 present the probabilistic sensitivity analysis results. Both the scatter plot of incremental costs and effects (Fig. 3) and the cost-effectiveness acceptability curve (Fig. 4) indicate that the probability of Pembro + FP being cost-effective was 0% at a WTP threshold of $50,000–$100,000/QALY.

**Table 3:** Results of scenario analyses on the selection of parametric function for OS and PFS

	Pembro + FP			FP			Incremental			ICER
	Cost($)	LY	QALY	Cost($)	LY	QALY	Cost($)	LY	QALY	($/QALY)
Base case (log logistic)	99,975.87	2.1	1.38	32,866.55	1.531	1	67,109.33	0.569	0.38	176,479
Exponential	97,202.77	1.783	1.23	30,699.58	1.342	0.9	66,503.18	0.441	0.33	205,780
Weibull	93,640.78	1.561	1.1	29,062.3	1.225	0.83	64,578.48	0.336	0.27	243,911
Gompertz	93,967.91	1.542	1.1	28,987.54	1.218	0.83	64,980.38	0.324	0.27	243,351
Log normal	100,227.92	2.055	1.36	32,031.82	1.458	0.96	68,196.1	0.597	0.4	169,784
Generalized gamma	97,373.59	1.739	1.22	30,151.76	1.31	0.88	67,221.83	0.429	0.34	200,237

*LY* life year, *QALY* quality—adjusted life year, *FP* fluoropyrimidine plus platinum-based chemotherapy, *Pembro + FP* Pembrolizumab plus FP therapy

## Discussion

This study evaluated the cost-effectiveness of Pembro + FP therapy versus FP therapy alone for unresectable advanced esophageal cancer from the perspective of Japanese healthcare payer. Based on a PartSA model, the ICER was $176,479 per QALY. In comparison with the WTP threshold of Japan, this ICER suggests that Pembro + FP therapy is not cost-effective, and sensitivity analyses supported this conclusion. The robustness of these findings was further demonstrated by subgroup analysis of patients with PD-L1 CPS ≥ 10, which also exceeded the WTP threshold of Japan.

Our study indicates that Pembro + FP therapy may not be cost-effective for unresectable advanced esophageal cancer patients in Japan. These findings partially differ from similar studies conducted in other countries. Wu et al. performed a cost-effectiveness analysis of Pembro + FP therapy for unresectable advanced esophageal cancer from the Chinese healthcare system perspective and estimated an ICER of USD 115,391 per QALY [8]. They reported that Pembro + FP therapy was not cost-effective compared to FP therapy alone when the WTP threshold was USD 31,304 per QALY [8]. They employed a Markov model with transition probabilities estimated from the Kaplan–Meier curves for OS and PFS in the KEYNOTE-590 trial. While they used utility values from the ToGA trial, as did our study, there was a substantial difference in the incremental QALY in the study of Wu et al. (2.32) versus our study (0.38) [8]. This discrepancy was attributable to differences in estimated life years. The Pembro + FP group had 5.36 estimated life years in the Wu et al. analysis compared with 2.10 estimated life years in our study, while the FP group had 3.71 versus 1.53 estimated life years, respectively [8]. Although we attempted to fit a log-logistic function to the curve-fitting results presented by Wu et al., we could not reproduce their findings, suggesting that their model specifications might not have been appropriate.

Qu et al. conducted a cost-effectiveness analysis of Pembro + FP therapy for unresectable advanced esophageal cancer using a PartSA model from the perspective of US healthcare payer and estimated an ICER of USD 147,097 per QALY [6]. They reported that Pembro + FP therapy was cost-effective compared to FP therapy alone when using a WTP threshold of USD 150,000 per QALY [6]. They performed parametric extrapolation using patient-level survival data from the KEYNOTE-590 trial [6]. In our study, as patient-level survival data from the KEYNOTE-590 trial were unavailable, we estimated individual patient survival data from the Kaplan–Meier curves and number of patients at risk reported in the KEYNOTE-590 trial. Both studies used log-logistic functions for long-term extrapolation. As a result, the estimated life years for the Pembro + FP groups were 2.27 years in the study of Qu et al. and 2.10 years in our study, while for the FP groups, they were 1.41 years and 1.53 years, respectively [6]. Although our estimated life years for the Pembro + FP group were less favorable compared to the study of Qu et al., our conclusions from the base-case analysis remained unchanged even when parameters were varied in favor of the Pembro + FP group in one-way sensitivity analyses, suggesting that uncertainty in survival estimation had a limited effect on our conclusions. In one-way sensitivity analyses, the ICER variations of Qu et al. were substantial, with multiple parameters resulting in ICERs exceeding USD 200,000 per QALY [6]. Furthermore, in their cost-effectiveness acceptability curve, the probability of being cost-effective at a WTP threshold of USD 150,000 was only 53%, barely above the reference value of 50% [6]. Considering the model uncertainty and sensitivity analysis results, it is difficult to assert the cost-effectiveness advantage of pembrolizumab based on the findings of Qu et al. In contrast, our sensitivity analyses showed consistent results regarding cost-effectiveness, demonstrating the robustness of our analysis.

Our study showed that in patients with PD-L1 CPS ≥ 10, the ICER was USD 126,862 per QALY, indicating better cost-effectiveness than the base-case analysis. In Japan, there are no use restrictions based on PD-L1 CPS. In the population with PD-L1 CPS ≥ 10, the Japanese subgroup demonstrated more favorable hazard ratios for OS (0.58 [95% CI 0.35–0.98]) and PFS (0.36 [95% CI 0.21–0.61]) compared with the overall population in the KEYNOTE-590 trial (OS: 0.62 [95% CI 0.49–0.78], PFS: 0.51 [95% CI 0.41–0.65]) [27]. Implementing PD-L1 CPS as a biomarker-based selection criterion in Japanese clinical practice could optimize the cost-effectiveness of this therapeutic strategy.

Drug pricing systems vary significantly across countries. The US allows pharmaceutical companies to set prices freely, increasing concerns about high drug costs, with the price of original anticancer drugs often rising even after generic versions become available [28]. The Chinese healthcare reimbursement framework is anchored by the National Reimbursement Drug List (NRDL), which incorporates a health technology assessment (HTA) alongside safety and efficacy in its evaluation criteria [29]. Their system allows drugs to be removed from the NRDL through medical technology reassessment, contributing to reducing healthcare costs [30]. Japan implemented its cost-effectiveness evaluation system in 2019 following a trial period from 2016 to 2018 [17]. Under this system, price adjustments based on ICERs are limited to partial price modifications. For specific items, including anticancer drugs, ICER thresholds are set at ¥7,500,000 per QALY, ¥11,250,000 per QALY, and ¥15,000,000 per QALY, with price reductions applied according to ICER values [16]. The Japanese drug pricing system incorporates price adjustment mechanisms through market expansion repricing for pharmaceuticals that exceed their projected annual sales thresholds. This recalculation affects both target drugs and similar listed drugs with identical pharmacological actions. Particularly, PD-1/PD-L1 inhibitors, with their broad range of cancer indications, are frequently subject to price revisions. In 2021, atezolizumab was subject to market expansion repricing, leading to a price reduction for pembrolizumab as a pharmacologically similar agent. Consequently, the cost of pembrolizumab in Japan became the lowest among major countries [12]. However, despite this price reduction, the cost-effectiveness of pembrolizumab for patients with unresectable advanced esophageal cancer in Japan remained suboptimal. Although a prior price reduction had been implemented, further substantial reductions would still be required to meet the WTP threshold. Our analysis revealed that pembrolizumab would fall below the WTP threshold if the drug cost was at or below USD 1240.78, requiring a 70% price reduction. Future price reductions in immune checkpoint inhibitors, including pembrolizumab, could potentially improve their cost-effectiveness by lowering the ICER. However, excessive price reductions could potentially exacerbate drug loss in Japan. In response to these concerns, the 2024 drug pricing system reform, considering the innovativeness of new drugs and impact on development incentives, resulted in a decision by the Central Social Insurance Medical Council to exempt certain medications, including PD-1/PD-L1 inhibitors, from similar-drug provisions in market expansion price adjustments. This study evaluated the cost-effectiveness of Pembro + FP therapy for unresectable advanced esophageal cancer during this transitional period in the pricing system. These findings provide valuable insights for healthcare policy discussions regarding pricing optimization strategies to enhance financial sustainability in healthcare systems comparable to that of Japan.

Compared with previous reports, our study presents several strengths. Notably, this is the first study to evaluate the cost-effectiveness of Pembro + FP therapy versus FP therapy alone for patients with unresectable advanced esophageal cancer from the perspective of Japanese healthcare payers using KEYNOTE-590 trial data. Furthermore, using the DPC database enabled analysis that reflects real-world medical costs in Japan. An economic evaluation chapter was recently added to the Minds Manual for Guideline Development, which is used in Japan [31]. From a healthcare resource allocation perspective, considering cost-effectiveness is expected to play an increasingly important role in future guideline development. This study provides valuable evidence for such guideline development initiatives.

There are some limitations in our study. First, the unavailability of EQ-5D data from the KEYNOTE-590 trial and the lack of alternative utility data for patients with esophageal cancer limited our utility data. However, previous studies also used similar utility values, assuming comparable utilities between esophageal and gastric cancers [32]. Therefore, we derived utility values from a previously published cost-effectiveness analysis of the ToGA trial [26]. We recognize that utility values for gastric cancer may not fully reflect those of esophageal cancer. Therefore, we performed sensitivity analyses by varying the utility values across plausible ranges. These analyses showed that our cost-effectiveness conclusions remained unchanged, suggesting the robustness of our results. Second, the cost estimates for outpatient treatment were limited because the DPC database does not include community pharmacy costs, potentially leading to an underestimation of the Cost. However, this limitation is unlikely to affect the conclusions of our cost-effectiveness analyses. This is because Pembro + FP therapy, with its longer survival time than FP therapy alone, would lead to higher subsequent treatment costs even when accounting for these additional expenses. Third, we excluded the costs of treating adverse events. This was justified by the Japanese subgroup analysis of the KEYNOTE-590 trial, which found comparable safety profiles between groups, including major adverse events such as neutropenia [33]. Fourth, there are concerns regarding potential confounding. Although the KEYNOTE-590 trial was randomized, patients enrolled in clinical trials may differ from those treated in routine clinical practice in terms of performance status and comorbidities. In addition, treatment selection in real-world settings is influenced by various factors, such as physician judgment and institutional practices, which may not be fully captured in our model. Treatment costs were estimated using real-world data from the DPC database in Japan. While the DPC data include information on activities of daily living (ADL) and comorbidities at admission, these variables were intentionally excluded from the model to maintain its simplicity and consistency. As a result, differences in patient backgrounds between treatment groups may have affected the estimated costs. Further research is needed to clarify how patient selection and treatment patterns influence the real-world cost-effectiveness of pembrolizumab plus chemotherapy.

In conclusion, the addition of pembrolizumab to FP therapy for unresectable advanced esophageal cancer resulted in an ICER above the WTP threshold in Japan.

## Supplementary Information

Below is the link to the electronic supplementary material.

Supplementary file1 (DOCX 23 KB)

Supplementary file2 (PPTX 104 KB)

## Data Availability

The datasets analyzed during the current study are available from the corresponding author on reasonable request.
