# Supplementary material for: Cost-effectiveness analysis of pembrolizumab plus chemotherapy versus chemotherapy as first line chemotherapy for patients with unresectable advanced esophageal cancer in Japan
Source: Esophagus. 2025 Jul 12;22(4):583–92. doi: 10.1007/s10388-025-01144-5 (PMC12450808; doi:10.1007/s10388-025-01144-5)
Supplement: Supplementary file 1 — Supplementary file1 (DOCX 23 KB) [file 10388_2025_1144_MOESM1_ESM.docx]

**Supplemental Table 1 AIC and BIC of curve fitting for OS and PFS in all population**

|  | OS in Pembro+FP group | | PFS in Pembro +FP group | | OS in FP group | | PFS in FP group | |
| --- | --- | --- | --- | --- | --- | --- | --- | --- |
|  | AIC | BIC | AIC | BIC | AIC | BIC | AIC | BIC |
| Exponential | 932.655 | 936.577 | 968.392 | 972.322 | 910.719 | 914.638 | 897.028 | 900.957 |
| Weibull | 911.887 | 919.730 | 939.290 | 947.149 | 892.229 | 900.067 | 838.778 | 846.637 |
| Gompertz | 928.551 | 936.395 | 961.746 | 969.605 | 912.434 | 920.271 | 882.242 | 890.102 |
| Log logistic | 897.230 | 905.073 | 921.331 | 929.190 | 845.943 | 853.781 | 812.055 | 819.914 |
| Log normal | 907.272 | 915.073 | 931.703 | 939.562 | 850.588 | 858.426 | 817.062 | 824.921 |
| Generalized gamma | 904.299 | 916.063 | 928.498 | 940.287 | 852.139 | 863.895 | 815.779 | 827.568 |

OS, Overall survival; PFS, Progression free survival; FP, fluoropyrimidine plus platinum-based chemotherapy; Pembro+FP, Pembrolizumab plus FP therapy; AIC, Akaike Information Criteria; BIC, Bayesian Information Criteria

**Supplemental Table 2 AIC and BIC of curve fitting for OS and PFS in PD-L1 CPS ≥ 10**

|  | OS in Pembro+FP group | | PFS in Pembro+ FP group | | OS in FP group | | PFS in FP group | |
| --- | --- | --- | --- | --- | --- | --- | --- | --- |
|  | AIC | BIC | AIC | BIC | AIC | BIC | AIC | BIC |
| Exponential | 483.232 | 486.458 | 496.614 | 499.840 | 529.275 | 532.558 | 493.661 | 496.944 |
| Weibull | 477.529 | 483.983 | 493.628 | 500.080 | 516.722 | 523.288 | 462.582 | 469.148 |
| Gompertz | 484.026 | 490.477 | 498.601 | 505.052 | 526.689 | 533.256 | 490.238 | 496.805 |
| Log logistic | 469.790 | 476.241 | 475.843 | 482.295 | 509.554 | 516.121 | 436.675 | 443.241 |
| Log normal | 473.271 | 479.723 | 478.696 | 485.147 | 513.578 | 520.145 | 446.575 | 453.141 |
| Generalized gamma | 473.982 | 483.66 | 480.506 | 490.183 | 512.485 | 522.334 | 445.158 | 455.008 |

OS, Overall survival; PFS, Progression free survival; FP, fluoropyrimidine plus platinum-based chemotherapy; Pembro+FP, Pembrolizumab plus FP therapy; AIC, Akaike Information Criteria; BIC, Bayesian Information Criteria

**Supplemental Table 3 Results of scenario analyses on the selection of parametric function for OS and PFS in PD-L1 CPS ≥ 10**

|  | Pembro+FP | |  |  | FP |  |  |  | Incremental |  |  |  | ICER |
| --- | --- | --- | --- | --- | --- | --- | --- | --- | --- | --- | --- | --- | --- |
|  | Cost($) | LY | QALY |  | Cost($) | LY | QALY |  | Cost($) | LY | QALY |  | ($/QALY) |
| Base case (log logistic) | 103,741.22 | 2.270 | 1.48 |  | 30,557.49 | 1.397 | 0.90 |  | 73,183.73 | 0.873 | 0.58 |  | 126,862 |
| Exponential | 97,439.16 | 1.833 | 1.25 |  | 28,106.45 | 1.188 | 0.79 |  | 69,332.71 | 0.645 | 0.46 |  | 151,025 |
| Weibull | 94,959.17 | 1.634 | 1.14 |  | 27,198.18 | 1.115 | 0.75 |  | 67,760.98 | 0.519 | 0.39 |  | 174,804 |
| Gompertz | 95,228.25 | 1.640 | 1.15 |  | 27032.07 | 1.105 | 0.75 |  | 68,196.17 | 0.535 | 0.40 |  | 167,543 |
| Log normal | 103070.15 | 2.205 | 1.45 |  | 29,505.35 | 1.302 | 0.85 |  | 73,564.80 | 0.903 | 0.60 |  | 123,495 |
| Generalized gamma | 98108.05 | 1.900 | 1.28 |  | 27,954.45 | 1.178 | 0.79 |  | 70,153.59 | 0.722 | 0.49 |  | 143,056 |

LY, life year; QALY, quality - adjusted life year; FP, fluoropyrimidine plus platinum-based chemotherapy; Pembro+FP, Pembrolizumab plus FP therapy
