# Supplementary material for: Cost-effectiveness analysis of pembrolizumab plus chemotherapy versus chemotherapy as first line chemotherapy for patients with unresectable advanced esophageal cancer in Japan
Source: Esophagus. 2025 Jul 12;22(4):583–92. doi: 10.1007/s10388-025-01144-5 (PMC12450808; doi:10.1007/s10388-025-01144-5)
Supplement: Supplementary file 2 — Supplementary file2 (PPTX 104 KB) [file 10388_2025_1144_MOESM2_ESM.pptx]

## Slide 1
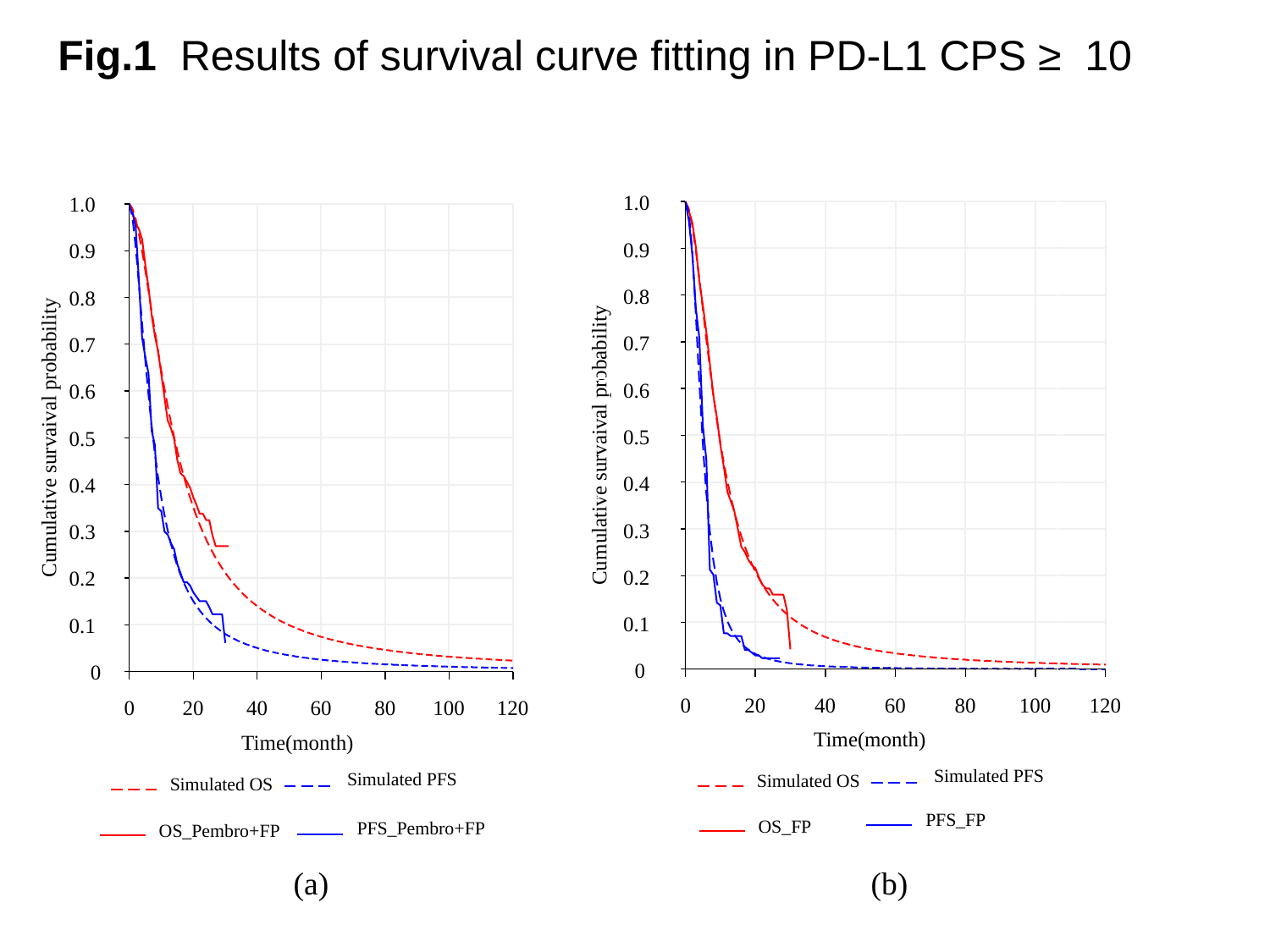

Fig.1 Results of survival curve fitting in PD-L1 CPS ≥ 10
1.0
0.9
0.8
0.7
0.6
0.5
Cumulative survaival probability
0.4
0.3
0.2
0.1
 0
0
20
40
60
80
100
120
Time(month)
1.0
0.9
0.8
0.7
0.6
0.5
0.4
0.3
0.2
0.1
 0
0
20
40
60
80
100
120
Time(month)
Cumulative survaival probability
Simulated PFS
Simulated PFS
Simulated OS
Simulated OS
PFS_FP
OS_FP
PFS_Pembro+FP
OS_Pembro+FP
(a)
(b)

## Slide 2
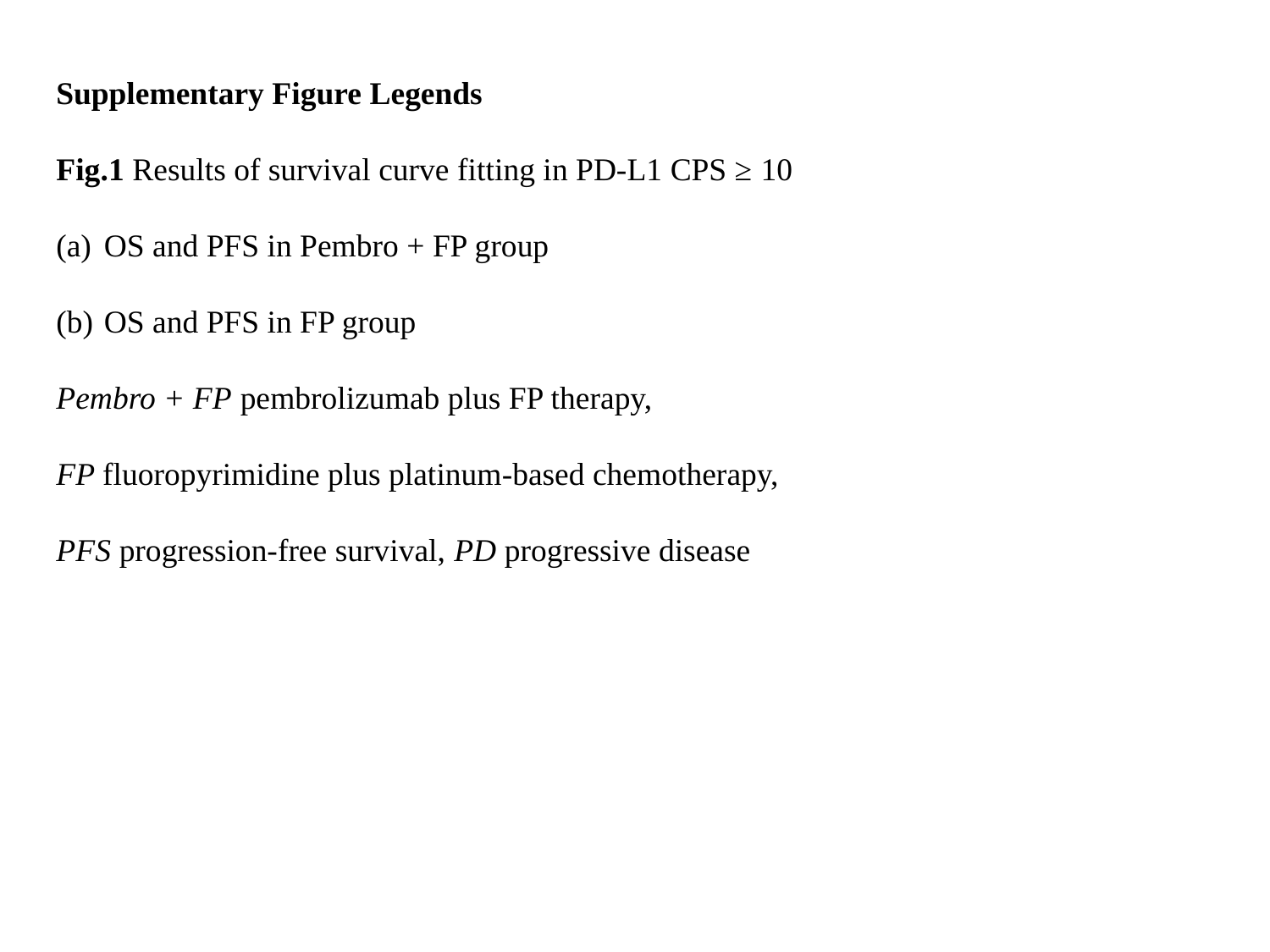

Supplementary Figure Legends
Fig.1 Results of survival curve fitting in PD-L1 CPS ≥ 10
OS and PFS in Pembro + FP group
OS and PFS in FP group
Pembro + FP pembrolizumab plus FP therapy,
FP fluoropyrimidine plus platinum-based chemotherapy,
PFS progression-free survival, PD progressive disease
